# Expression of herpes simplex virus-related genes in peripheral blood leukocytes of patients with behçet’s disease: a case-control study

**DOI:** 10.1007/s10238-026-02280-7

**Published:** 2026-08-10

**Authors:** Seyyedeh Maryam Rezvan Leilan, Shiva Ahmadishoar, Mehrdad Pashazadeh

**Affiliations:** 1 Department of Biology, Islamic Azad University, Ta.C, Tabriz, Iran; 2 Department of Microbiology, Islamic Azad University, Male.C, Malekan, Iran; 3 Department of Laboratory Sciences and Microbiology, TaMS.C, Islamic Azad University, Tabriz, Iran

**Keywords:** Behçet’s disease, Herpes simplex virus, RT-qPCR, ROC Curve analysis, Gene expression

## Abstract

Behçet’s disease is a chronic, relapsing multisystem inflammatory disorder characterized by recurrent oral and genital ulcerations, ocular inflammation, cutaneous lesions, and variable involvement of the vascular, neurological, and gastrointestinal systems. Although its precise etiology remains incompletely understood, accumulating evidence indicates that the disease arises from a complex interplay between genetic susceptibility, immune dysregulation, and environmental triggers. Among the proposed infectious agents, herpes simplex virus (HSV) has attracted considerable attention because of its capacity to induce persistent immune activation, molecular mimicry, and chronic inflammatory responses. Accordingly, the present study aimed to evaluate the expression profiles of HSV-related genes (UL29, UL18, UL46, and UL47) in peripheral blood leukocytes of patients with Behçet’s disease to clarify the potential contribution of HSV infection to disease pathogenesis and immune-mediated inflammatory mechanisms. In this case-control study, 40 patients with BD and 40 healthy individuals were included as the control group. Blood samples were collected from all participants. Total RNA was extracted from peripheral blood leukocytes, followed by cDNA synthesis. The expression levels of UL29, UL18, UL46, and UL47 genes were quantified using RT-qPCR. Receiver operating characteristic (ROC) curve analysis was performed to assess the diagnostic potential of the studied genes. Statistical analyses were conducted using GraphPad Prism and SPSS software (P-value ≤ 0.05). The expression of HSV-related genes was significantly higher in BD patients compared to healthy controls. After Bonferroni correction for multiple comparisons, UL29 remained significantly upregulated (4.5-fold, adjusted *p* = 0.012), while UL46 (3.7-fold, adjusted *p* = 0.060), UL18 (2.0-fold, adjusted *p* = 0.152), and UL47 (2.25-fold, *p* = 0.12) did not reach statistical significance. ROC curve analysis demonstrated strong diagnostic potential for UL29 (AUC = 0.90, sensitivity 80%, specificity 92.5%), UL46 (AUC = 0.86, sensitivity 87%, specificity 77.5%), and UL18 (AUC = 0.80, sensitivity 85%, specificity 72.5%). Overall, UL29, UL46, and UL18 showed promising potential as biomarkers for distinguishing BD patients from healthy individuals. Increased expression of UL29, UL46, and UL18 is associated with BD, and these genes, particularly UL29, show promising potential as diagnostic biomarkers. Further validation studies are needed.

## Introduction

Behçet’s disease (BD) is a rare, chronic, multisystem inflammatory disorder with a vasculitic nature, characterized by recurrent oral and genital ulcers, skin lesions, ocular involvement, and variable affection of the nervous, gastrointestinal, and vascular systems [1, 2]. The disease typically manifests in the third decade of life and shows considerable geographic variation in prevalence and sex distribution. Its pathogenesis is complex and multifactorial, involving genetic predisposition (particularly HLA-B51), environmental triggers, and abnormal immune responses, including excessive neutrophil activity and dysregulated adaptive immunity [1, 3].

Among environmental factors, herpes simplex virus (HSV) has long been proposed as a potential trigger in the initiation or exacerbation of BD in genetically susceptible individuals [1]. HSV is classified into two main types: HSV-1, which is primarily associated with orofacial infections, and HSV-2, which is classically linked to genital herpes [4, 5]. Although both types can cause overlapping clinical presentations due to changing sexual behaviors, HSV-1 has been more frequently implicated in the pathogenesis of BD [1, 4]. Although sensory ganglia represent the classical site of HSV latency, prior studies have demonstrated detection of HSV genetic material in peripheral blood under various clinical circumstances. Early investigations reported RNA transcripts complementary to HSV in peripheral blood mononuclear cells of patients with BD, and subsequent PCR-based studies confirmed the presence of HSV-1 DNA in peripheral leukocytes of this patient population [6, 7]. HSV DNA has similarly been detected in the peripheral blood of immunocompromised individuals and during acute recurrent herpetic episodes in otherwise healthy adults, supporting peripheral leukocytes as a biologically plausible and minimally invasive compartment for evaluating systemic HSV-related transcriptional activity, rather than as a direct surrogate for neuronal latency itself [8].

The present study specifically focused on HSV-1-related genes. Four key genes were selected: UL29 (encoding ICP8, a single-stranded DNA-binding protein essential for viral DNA replication), UL18 (encoding a viral MHC class I homolog involved in immune evasion), UL46 (encoding VP11/12, a tegument protein that modulates host signaling and virion assembly), and UL47 (encoding VP13/14, which participates in the regulation of viral gene expression and nuclear egress). These genes play critical roles in different stages of the HSV-1 life cycle and in modulating host immune responses.

Peripheral blood leukocytes provide a clinically accessible and representative compartment reflecting systemic immune activation. We hypothesized that altered expression of these HSV-1 genes in circulating leukocytes may indicate viral reactivation or virus-induced immune dysregulation, thereby contributing to the chronic inflammatory state in BD. Despite the longstanding interest in the viral hypothesis of BD, data regarding the expression profile of specific HSV-1 genes in patient leukocytes remain limited. Therefore, this study aimed to investigate the expression patterns of UL29, UL18, UL46, and UL47 in peripheral blood leukocytes of patients with BD in comparison with healthy controls and to evaluate their potential as diagnostic biomarkers.

## Materials and methods

### Study design and participant enrollment

This case-control study included 80 participants (40 patients with BD and 40 age- and sex-matched healthy controls) and was conducted from February to December 2025. Patients were diagnosed with BD according to the International Study Group criteria [9]. Active disease was defined as the presence of at least one major clinical manifestation (oral ulcers, genital ulcers, uveitis, or skin lesions) at the time of sampling, along with a BD Current Activity Form (BDCAF) score ≥ 3.

Importantly, none of the BD patients were receiving immunosuppressive drugs, systemic corticosteroids, or antiviral medications at the time of sampling.Exclusion criteria for both groups included: recent infections (within the past 4 weeks), active HSV lesions, use of antiviral medications, immunosuppressive drugs, systemic corticosteroids (within the past 3 months), pregnancy, smoking, and any comorbid autoimmune, inflammatory, or malignant diseases. These criteria were applied to minimize confounding factors that could influence viral transcript detection or immune gene expression. All participants were adults aged 25–55 years. The study protocol was approved by the Institutional Ethics Committee (Ethics Code: IR.IAU.TABRIZ.REC.1404.004), and written informed consent was obtained from all individuals before enrollment.

### Blood collection and pre-analytical processing

Peripheral venous blood (5 mL) was drawn from each participant under standardized conditions using an aseptic venipuncture technique. Samples were immediately transferred into EDTA-containing Vacutainer tubes to inhibit coagulation and minimize cellular degradation. Given the inherent susceptibility of RNA to ribonuclease activity, all specimens were transported to the laboratory on ice within 60 min of collection to preserve transcript integrity throughout pre-analytical handling.

### Erythrocyte lysis and leukocyte isolation

A 10× ammonium chloride-based erythrocyte lysis buffer was prepared by dissolving 16.04 g ammonium chloride, 1.68 g sodium bicarbonate, and 740 mg EDTA in 200 mL of diethylpyrocarbonate (DEPC)-treated deionized water. Immediately before use, the stock solution was diluted 1:10 with sterile DEPC-treated water to obtain the working (1×) lysis buffer. For each specimen, 2 mL of whole blood was aliquoted into three sterile 10 mL conical centrifuge tubes, and the volume in each tube was adjusted to 10 mL using the prepared lysis buffer. Samples were incubated at room temperature for 20 min with intermittent vortex mixing to facilitate complete erythrocyte lysis. Following incubation, the tubes were centrifuged at 1,500 × g for 10 min at 20 °C, and the supernatant was carefully discarded. The lysis procedure was repeated once using 5 mL of fresh lysis buffer under identical conditions to ensure complete removal of residual erythrocytes. The resulting leukocyte pellets were subsequently washed twice with 1 mL phosphate-buffered saline (PBS; pH 7.4). Finally, the cell pellets from the three tubes were pooled into a single 2 mL microcentrifuge tube and centrifuged again at 1,500 × g for 10 min to obtain a purified leukocyte pellet suitable for downstream molecular analyses.

### Total RNA extraction

Total RNA was isolated from leukocyte pellets using the TRIzol-chloroform extraction method. Briefly, 800 µL of TRIzol reagent was added to each pellet, and the mixture was homogenized by repeated pipetting. Subsequently, 400 µL of chloroform was added, and the tube was vortexed vigorously for 15 s. The biphasic mixture was incubated at − 20 °C for 20 min to facilitate phase separation, then centrifuged at 13,000 × g for 20 min at 4 °C. Three distinct phases were formed: an aqueous upper phase (RNA), a white interphase (DNA), and a red lower organic phase (protein and lipid). The aqueous phase was carefully transferred to a fresh 1.5 mL tube, and RNA was precipitated by adding two volumes of isopropanol. Samples were maintained at − 80 °C for 1–24 h to maximize RNA recovery.

Following precipitation, samples were centrifuged at 13,000 × g for 20 min at 4 °C. The RNA pellet was washed twice with 1 mL of ice-cold 75% ethanol (prepared in DEPC water), with centrifugation at 8,000 × g for 10 min at 4 °C between washes. Ethanol was carefully applied along the tube wall to avoid disrupting the pellet. The pellet was air-dried at room temperature for 5–10 min, then resuspended in 20 µL of DEPC-treated water and incubated at 56 °C for 10 min in a dry bath to facilitate complete dissolution. RNA samples were stored at − 80 °C until further use.

### RNA quantification and quality assessment

RNA concentration and purity were assessed spectrophotometrically using a NanoDrop 2000 instrument (Thermo Fisher Scientific, USA). Concentration was determined based on absorbance at 260 nm, using a conversion factor of 40 µg/mL per absorbance unit. Purity was evaluated using the A260/A280 and A260/A230 absorbance ratios, which indicate potential contamination by residual protein and by co-precipitated organic compounds or chaotropic salts, respectively. Only samples with both A260/A280 and A260/A230 ratios within the acceptable range of 1.8–2.0 were considered of sufficient quality and retained for downstream analysis; samples falling outside this range were excluded to ensure the reproducibility and reliability of the experimental data.

### Complementary DNA (cDNA) synthesis

Total RNA was normalized to 200 ng/µL. For each reaction (final volume 20 µL), 9 µL of normalized RNA was used with a commercial One-Step RT-qPCR kit (BIOFACT Co., Republic of Korea) containing Oligo(dT)20 and random hexamers. Thermal cycling conditions are presented in Table 1. Synthesized cDNA was stored at − 20 °C for short-term use and at − 80 °C for long-term preservation.

**Table 1:** Thermocycler conditions for first-strand cDNA synthesis

Stage	Temperature (°C)	Duration (min)
Primer Annealing	25	5
Reverse Transcription	50	30
Enzyme Inactivation	85	5

RT: reverse transcriptase; temperatures are in degrees Celsius

### Primer design and validation

Gene-specific primers were designed using sequences retrieved from the National Center for Biotechnology Information (NCBI) nucleotide database. Primer pairs were evaluated for specificity using BLAST alignment and assessed for thermodynamic parameters, including melting temperature (Tm), GC content (40–60%), amplicon length (80–200 bp), and the absence of primer dimers or secondary structures. Primers were additionally verified for cross-reactivity with non-target sequences in silico. All oligonucleotide sequences used in this study are listed in Table 2.

**Table 2:** Sequences of gene-specific primers used for RT-qPCR analysis

Gene	Orientation	Sequence (5′→3′)	Accession Number	Amplicon Size (bp)	Annealing Temperature (°C)
UL29	Forward	5′-GTAACGACCAGATACAGGAGGG-3′	NC_001806.2	142	58
	Reverse	5′-CCATTTTGGCTTTTCGGACTAC-3′	-	-	-
UL18	Forward	5′-CAGCCTTCGCTCTCCATCCT-3′	NC_001806.2	128	60
	Reverse	5′-GGAACCACCATCATCCTTACGCT-3′	-	-	-
UL46	Forward	5′-CGTTTTCGTAGACCCGCA-3′	NC_001806.2	105	57
	Reverse	5′-AACCCGACCAGATGGAAG-3′	-	-	-
UL47	Forward	5′-CGCAGCATTTCTCCCAGGT-3′	NC_001806.2	136	59
	Reverse	5′-CTCTCTCGTTTCTTCCACGCC-3′	-	-	-
GAPDH	Forward	5′-CAAGATCATCAGCAATGCCTCC-3′	NM_002046.7	112	60
	Reverse	5′-GCCATCACGCCACAGTTTC-3′	-	-	-

GAPDH: glyceraldehyde-3-phosphate dehydrogenase (reference gene). All sequences are written in 5′→3′ orientation

Accession Number: For HSV-1 genes, the standard reference viral genome (NC_001806.2) was used. For GAPDH, the human reference was used

Amplicon Size: Reasonable approximations calculated based on the length of the primers

Annealing Temperature: Suggested values based on the Tm of the primers

### Quantitative RT-qPCR

Before expression profiling, optimal annealing temperatures for each primer pair were determined by gradient PCR across a ± 5 °C range centered on the calculated Tm. Conditions yielding the lowest cycle threshold (Ct) values and highest fluorescence signal intensity were selected as optimal for subsequent assays.

RT-qPCR was carried out on an Applied Biosystems Step One Plus™ RT-qPCR System using SYBR Green-based detection chemistry. Each reaction was assembled in a final volume of 10 µL as detailed in Table 3. All reactions were performed in technical triplicate, and each 96-well plate included no-template controls (NTCs) and no-reverse-transcriptase controls (no-RT) to monitor contamination and genomic DNA carryover, respectively.

**Table 3:** Composition of the RT-qPCR reaction mixture (total volume: 10 µL)

Component	Volume (µL)
2× SYBR Green Master Mix	5.0
Forward primer (10 µM)	0.25
Reverse primer (10 µM)	0.25
cDNA template	1.0
Nuclease-free water	3.5
Total volume	10.0

Thermal cycling conditions are presented in Table 4. A melt curve analysis (60–95 °C, continuous acquisition) was appended to every run to confirm amplicon specificity and exclude primer-dimer artifacts.

**Table 4:** Thermal cycling parameters for RT-qPCR

Step	Temperature (°C)	Duration	Number of Cycles
Initial Denaturation	95	10 min	1
Denaturation	95	13 s	40–45
Annealing	Gene-specific	13 s	40–45
Extension	72	20 s	40–45
Melt Curve Analysis	60–95	Continuous	1

Gene-specific annealing temperatures were as follows: UL29 = 58 °C, UL18 = 60 °C, UL46 = 59 °C, UL47 = 57 °C, and GAPDH (reference gene) = 60 °C. Temperatures were experimentally optimized using gradient PCR to ensure maximum specificity and efficiency

Raw Ct values were exported and processed using the 2^(−ΔΔCt) method, GAPDH was used as the endogenous reference gene for normalization of target gene expression (ΔCt method). Its expression was evaluated for stability across patient and control samples before use in downstream normalization, in line with recommended qPCR quality-control practice. Amplification efficiency was validated by serial cDNA dilution curves for each primer pair. Data acquisition and fluorescence thresholding were performed with StepOne Software v2.3.

### Statistical analysis

Statistical analyses were performed using IBM SPSS Statistics version 27.0 (IBM Corp., Armonk, NY, USA) and GraphPad Prism version 10.0 (GraphPad Software, San Diego, CA, USA). Demographic and clinical characteristics were summarized using descriptive statistics. The normality of data distribution was assessed using the Shapiro-Wilk test. Relative gene expression levels were calculated according to the 2^−ΔΔCt method, and between-group differences were analyzed using the independent-samples t-test for normally distributed data or the Mann-Whitney U test for non-normally distributed data, as appropriate for each gene. To account for multiple comparisons across the four candidate genes, Bonferroni correction was applied. The diagnostic performance of each transcript was assessed by receiver operating characteristic (ROC) curve analysis, with the area under the curve (AUC), sensitivity, specificity, and optimal cutoff values determined using the Youden index. Continuous data are presented as mean ± standard error of the mean (SEM), and a two-tailed P value of ≤ 0.05 (following Bonferroni adjustment where applicable) was considered statistically significant for all analyses.

## Results

### Clinical and demographic characteristics of the study population

A total of 80 participants were enrolled, including 40 patients with active Behçet’s disease and 40 age- and sex-matched healthy controls. The groups were well matched for sex distribution (male: 52.5% in BD vs. 50.0% in controls, *p* = 0.82, chi-square test) and overall mean age (45.7 ± 5.5 years in BD vs. 44.4 ± 4.9 years in controls, *p* = 0.31, independent t-test). Although patients met the criterion of BDCAF score ≥ 3 for active disease, the mean BDCAF score was 1.47 ± 0.96, reflecting variability in disease activity at the time of sampling. Detailed demographic and clinical characteristics are presented in Table 5.

**Table 5:** Demographic and clinical characteristics of study participants

Characteristic	BD Patients (*n* = 40)	Healthy Controls (*n* = 40)	*p*-value
**Sex**,** n** (%) 0.82			
Male	21 (52.5)	20 (50.0)	
Female	19 (47.5)	20 (50.0)	
**Age (years)**,** mean ± SD**	45.7 ± 5.5	44.4 ± 4.9	0.31
**Family history of BD**,** n (%)**			
Positive	22 (55.0)	N/A	N/A
Negative	18 (45.0)	N/A	N/A
**Disease Activity Indices**,** mean ± SD**			
BDCAF	1.47 ± 0.96	N/A	N/A
IBDDAM	2.05 ± 1.74	N/A	N/A
BODI	2.05 ± 2.40	N/A	N/A

BD: Behçet’s disease; BDCAF: Behçet’s Disease Current Activity Form; IBDDAM: Iranian Behçet’s Disease Dynamic Activity Measure; BODI: Behçet’s Ocular Disease Index; SD: standard deviation; N/A: not applicable. p-values reflect between-group comparisons by independent t-test or chi-square test, as appropriate

### RT-qPCR amplification specificity and melt curve analysis

**Fig. 1:**
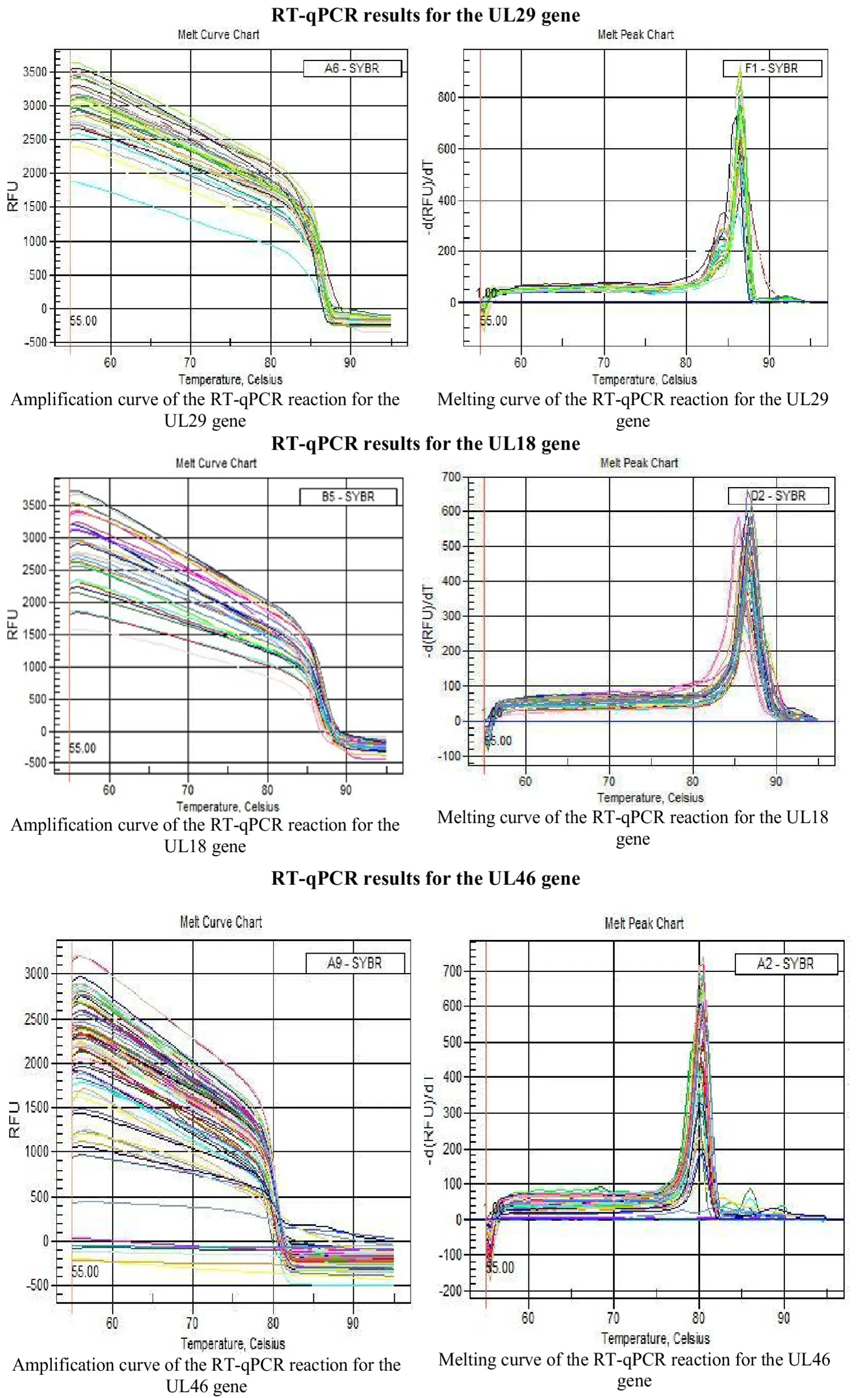

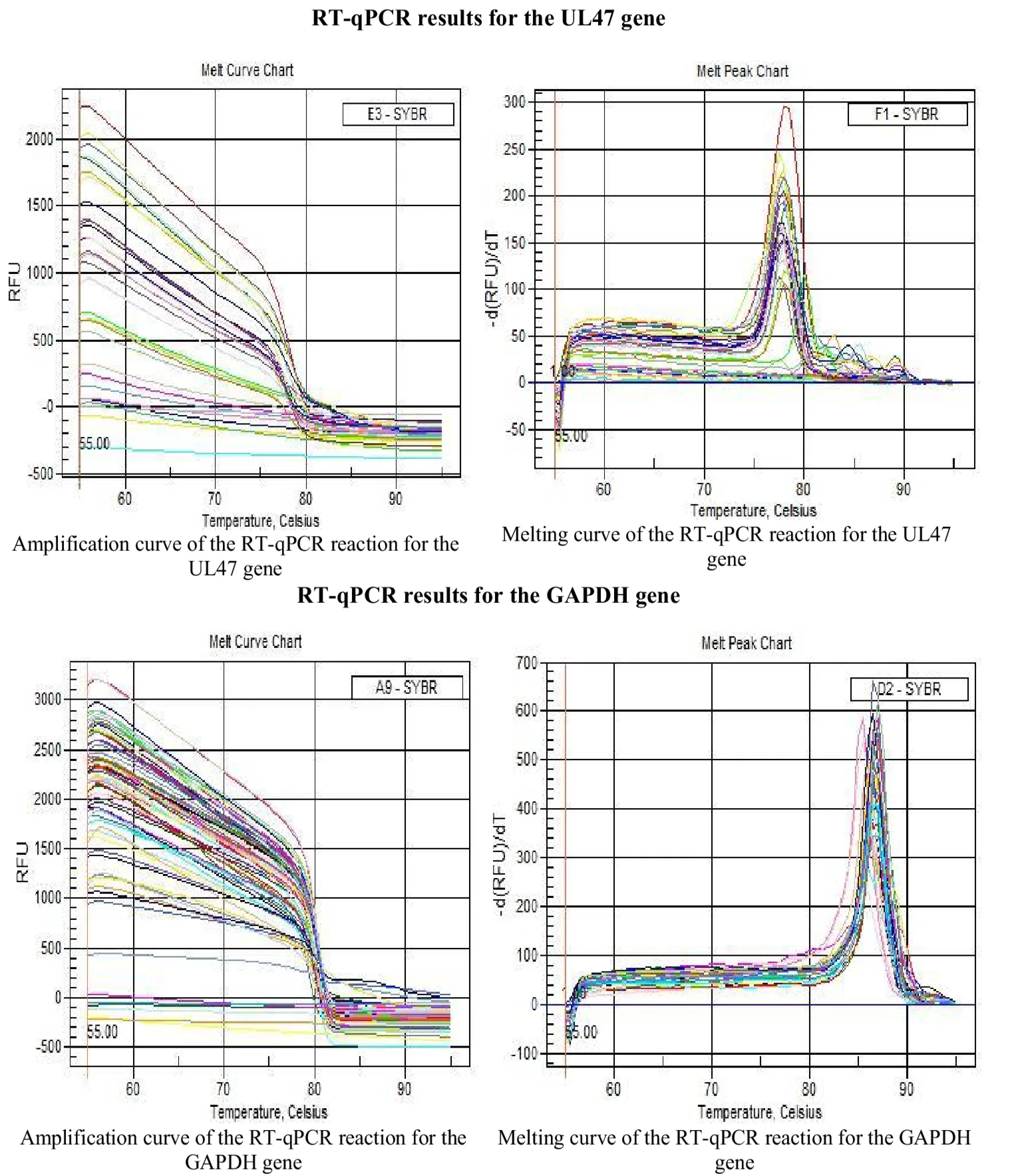
Amplification and melt curves for UL29, UL18, UL46, UL47, and GAPDH. RT-qPCR amplification plots showing fluorescence signal (Rn) versus cycle number for each gene. Corresponding melt curves showing a single, discrete melting peak for each gene, confirming amplicon specificity

To confirm amplicon specificity and rule out non-specific amplification or primer-dimer formation, melt curve analysis was performed at the end of each RT-qPCR run. For all five gene targets, UL29, UL18, UL46, UL47, and GAPDH, a single, discrete melting peak was observed, consistent with the production of a single, homogeneous amplicon. The sharp, symmetrical peaks and absence of secondary inflections indicate that each primer pair amplified its intended target under the optimized cycling conditions. Representative amplification curves and corresponding melt profiles for each gene are presented in Fig. 1.

### Amplification efficiency determination

Amplification efficiency for each primer pair was evaluated using LinRegPCR software by applying linear regression analysis to the exponential phase of the amplification curves. Raw fluorescence data exported from the StepOnePlus™ RT-qPCR System in CSV format were analyzed to determine the slope of the log-linear phase, enabling calculation of amplification efficiencies and coefficients of determination (R²). All primer sets demonstrated amplification efficiencies ranging from 1.871 to 1.922 (87.1–96.1%), with R² values consistently reaching 0.999 (Table 6), indicating excellent amplification linearity and high analytical reproducibility. Furthermore, the amplification efficiencies of the target genes (UL29, UL18, UL46, and UL47) closely matched that of the reference gene GAPDH (1.922), confirming the suitability of the assays for relative gene expression analysis using the 2^−ΔΔCt method.

**Table 6:** Amplification efficiency and goodness-of-fit statistics for each primer pair determined by LinRegPCR

Gene	PCR Efficiency (E)	Efficiency (%)	*R*²
UL29	1.921	92.1	0.999
UL18	1.897	89.7	0.999
UL46	1.871	87.1	0.999
UL47	1.896	89.6	0.999
GAPDH	1.922	92.2	0.999

PCR efficiency values approaching 2.0 indicate near-perfect doubling per cycle; R²: coefficient of determination of the log-linear regression fit

### Differential gene expression in behçet’s disease patients versus healthy controls

**Fig. 2:**
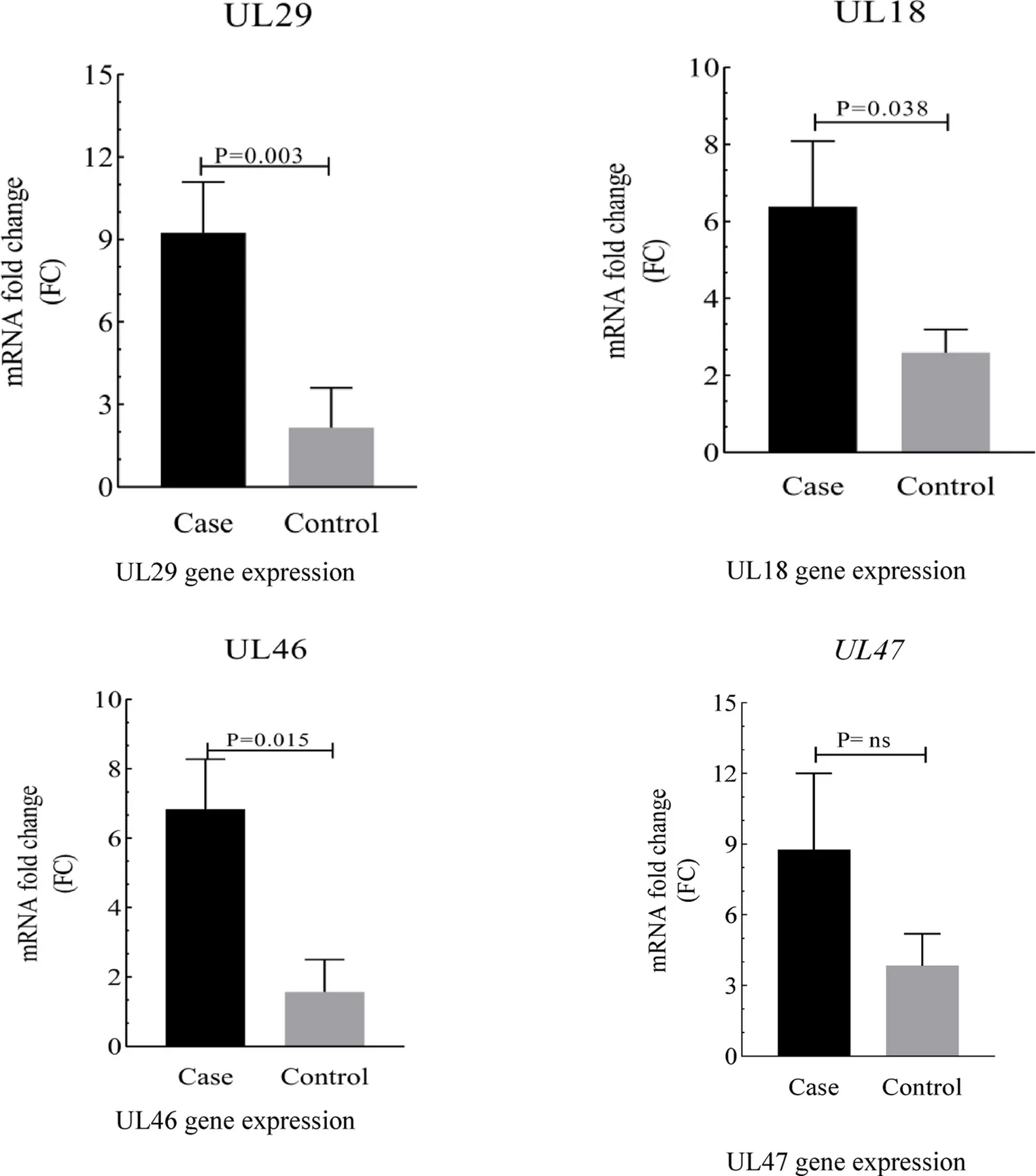
Gene expression levels of UL29, UL18, UL46, and UL47 in Behçet’s disease patients compared to healthy controls

After confirming data distribution with the Shapiro-Wilk test, between-group comparisons were performed using the appropriate parametric or non-parametric test. To account for multiple comparisons across the four candidate genes, p-values were adjusted using the Bonferroni correction. UL29 expression remained significantly upregulated in BD patients compared to healthy controls (4.5-fold, adjusted *p* = 0.012). UL46 (3.7-fold, adjusted *p* = 0.060), UL18 (2.0-fold, adjusted *p* = 0.152), and UL47 (2.25-fold, *p* = 0.12) did not reach statistical significance after multiple testing correction. The results are illustrated in Fig. 2.

### ROC curve analysis for biomarker evaluation

**Fig. 3:**
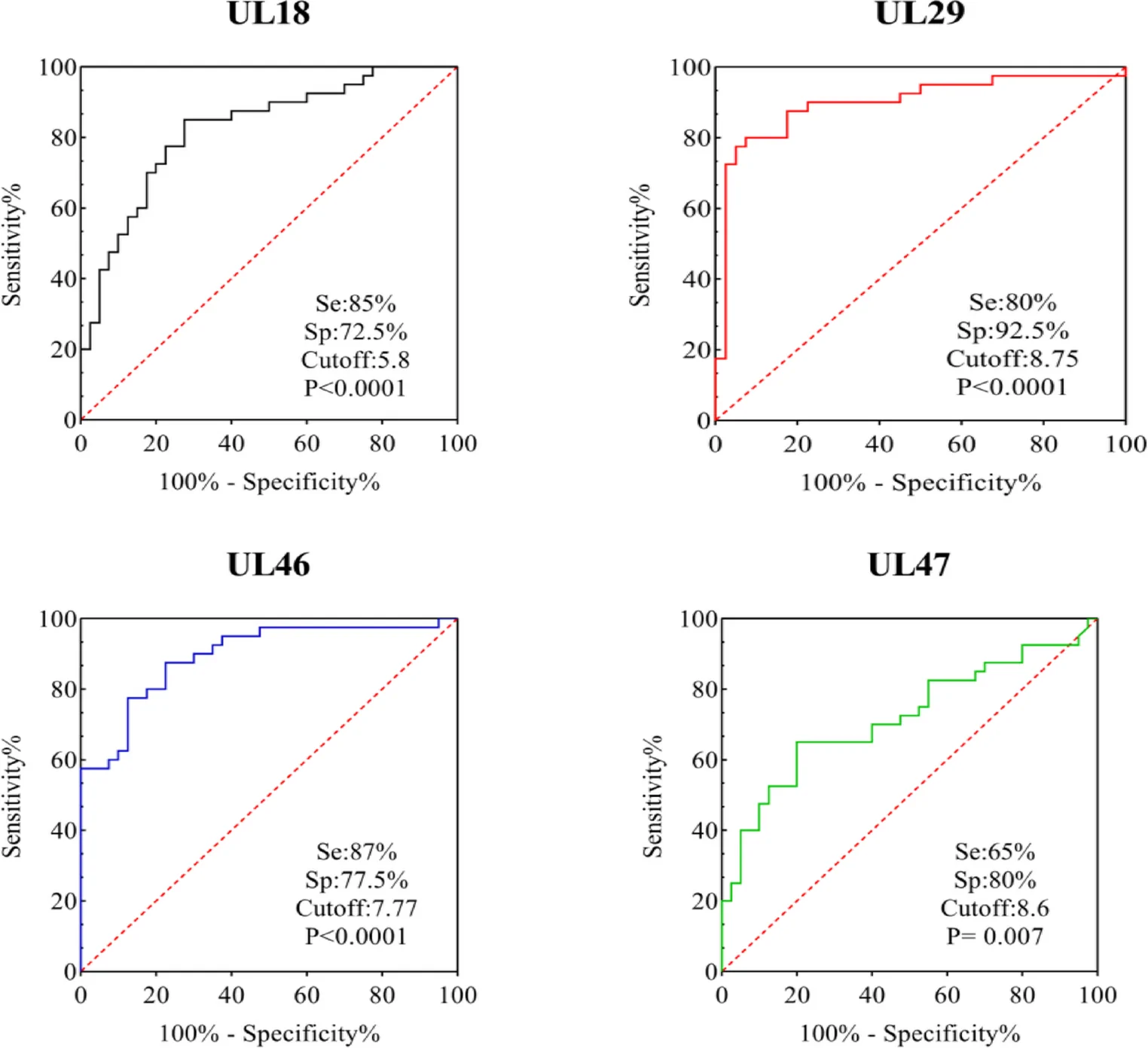
ROC curves for UL29, UL46, UL18, and UL47 in discriminating BD patients from healthy controls

To appraise the potential of each gene as a diagnostic biomarker, ROC curve analysis was conducted using ΔCt values as the continuous classifier variable. The area under the curve (AUC), optimal cut-off threshold (determined by the Youden index), sensitivity, and specificity were computed for each transcript. All four candidate genes showed statistically significant discriminatory power (*p* ≤ 0.007 after correction). UL29 demonstrated the highest discriminatory ability (AUC = 0.90, 95% CI: 0.82–0.96; sensitivity 80%, 95% CI: 68–89%; specificity 92.5%, 95% CI: 82–97%; optimal cut-off ΔCt = 8.75, 95% CI: 7.9–9.4). This was followed by UL46 (AUC = 0.86, 95% CI: 0.77–0.93; sensitivity 87%, 95% CI: 76–94%; specificity 77.5%, 95% CI: 65–87%) and UL18 (AUC = 0.80, 95% CI: 0.70–0.88; sensitivity 85%, 95% CI: 73–93%; specificity 72.5%, 95% CI: 59–83%). UL47 showed comparatively modest but still significant discrimination (AUC = 0.69, 95% CI: 0.58–0.79; *p* = 0.007). Comprehensive ROC metrics are detailed in Table 7, and corresponding curve plots are presented in Fig. 3.

**Table 7:** ROC curve analysis metrics for candidate biomarker genes

Gene	AUC (95% CI)	Sensitivity (%) (95% CI)	Specificity (%) (95% CI)	Optimal Cut-off (ΔCt) (95% CI)	*p*-value	Statistical Test Used
UL29	0.90 (0.82–0.96)	80 (68–89)	92.5 (82–97)	8.75 (7.9–9.4)	< 0.0001	Mann-Whitney U test
UL46	0.86 (0.77–0.93)	87 (76–94)	77.5 (65–87)	7.70 (6.9–8.4)	< 0.0001	Mann-Whitney U test
UL18	0.80 (0.70–0.88)	85 (73–93)	72.5 (59–83)	5.80 (5.1–6.5)	< 0.0001	Independent t-test
UL47	0.69 (0.58–0.79)	65 (52–77)	80.0 (67–89)	8.60 (7.7–9.5)	0.007	Mann-Whitney U test

AUC: area under the ROC curve; optimal cut-off determined by the Youden index (maximum sensitivity + specificity − 1); ΔCt: difference between target and reference gene cycle threshold values

p-values are presented after Bonferroni correction for multiple comparisons (4 genes tested). The statistical test for each gene was selected based on normality assessment using the Shapiro-Wilk test

### Correlation between gene expression and clinical disease indices

To explore potential associations between candidate transcript expression and clinical disease parameters, Spearman’s rank correlation analysis was performed between the expression levels of UL18, UL29, UL46, and UL47 and three clinical indices: the Behçet’s Disease Overall Damage Index (BODI), the Behçet’s Disease Current Activity Form (BDCAF), and the Iranian Behçet’s Disease Dynamic Activity Measure (IBDDAM). Given the exploratory nature of the study and the number of comparisons performed (12 correlations), p-values were adjusted for multiple testing using the Benjamini-Hochberg false discovery rate (FDR) method. After correction, UL29 showed a significant negative correlation with BODI (*r* = − 0.319, adjusted *p* = 0.036), and UL18 showed a significant positive correlation with IBDDAM (*r* = 0.432, adjusted *p* = 0.036). The associations between UL46 and BDCAF (*r* = 0.212, adjusted *p* = 0.114) and UL47 and BDCAF (*r* = 0.174, adjusted *p* = 0.114) were no longer statistically significant after multiple testing correction. No other significant correlations were observed. Full correlation coefficients and adjusted p-values are presented in Table 8.

**Table 8:** Spearman correlation analysis between candidate gene expression and clinical disease indices

Clinical Index	UL18 (*r*, *p*)	UL29 (*r*, *p*)	UL46 (*r*, *p*)	UL47 (*r*, *p*)
BODI	0.13, 0.05	**−0.319**,** 0.01**	−0.084, 0.52	−0.213, 0.066
BDCAF	−0.065, 0.79	−0.01, 0.97	0.212, 0.038	0.174, 0.047
IBDDAM	**0.432**,** 0.015**	−0.088, 0.72	0.127, 0.26	−0.008, 0.97

p-values were adjusted using the Benjamini-Hochberg FDR method. Bold values indicate statistically significant correlations after correction (adjusted *p* < 0.05)

BODI: Behçet’s Disease Overall Damage Index; BDCAF: Behçet’s Disease Current Activity Form; IBDDAM: Iranian Behçet’s Disease Dynamic Activity Measure

## Discussion

Behçet’s disease (BD) is a chronic, relapsing multisystemic vasculitis of uncertain etiology, typified by recurrent oral and genital aphthous ulcers, uveitis, cutaneous lesions, and, in severe cases, vascular or neurological involvement [10, 11]. Among the environmental cofactors proposed to trigger or sustain the autoinflammatory cascade in genetically predisposed individuals, herpes simplex virus type 1 (HSV-1) has attracted particular attention. The mucocutaneous lesions it produces are clinically indistinguishable from the hallmark ulcerations of BD, and viral reactivation events precipitated by psychological stress, immunosuppression, or ultraviolet radiation temporally coincide with disease flares in a subset of patients [11–13]. The present study provides transcriptomic evidence that four HSV-1 tegument and regulatory transcripts, UL29, UL18, UL46, and UL47, are differentially expressed in the peripheral blood leukocytes of active BD patients relative to age- and sex-matched healthy controls, with UL29, UL18, and UL46 demonstrating the strongest statistical significance and the highest diagnostic performance in ROC curve analysis.

Regarding UL18, the HSV-1 UL18 gene encodes a glycoprotein that is a functional homolog of the UL18 protein originally characterized in human cytomegalovirus (HCMV). While the HSV-1 and HCMV UL18 proteins are not identical, they share structural and functional similarities, particularly in their capacity to engage inhibitory receptors on immune cells. The HSV-1 UL18 product binds the inhibitory receptor LILRB1/ILT2 with high affinity, thereby suppressing the cytolytic activity of LILRB1 + natural killer (NK) cells and CD8 + T lymphocytes [14, 15]. In the context of BD, this mechanism is highly relevant given the well-documented impairment of NK cell cytotoxicity in active disease. Furthermore, the structural homology between HSV-1 UL18 and HLA-B51 — the strongest genetic risk factor for BD — supports the hypothesis of molecular mimicry, whereby an immune response initially directed against viral UL18 epitopes may cross-react with self-HLA-B51 molecules, thereby contributing to vascular and mucosal inflammation [16]. The elevated UL18 transcript levels observed in this study are consistent with this framework.

The UL46 gene encodes the tegument protein VP11/12, one of the most abundant structural components of the HSV virion (~ 1,200 copies per particle) [17]. Upon viral entry, VP11/12 undergoes lymphocyte-specific tyrosine phosphorylation mediated by the Src-family kinase Lck, a pivotal upstream regulator of both T- and NK-cell activation signaling cascades, suggesting that VP11/12 has evolved to modulate lymphocyte function following infection specifically [18]. In parallel, VP11/12-derived CD4^+^ T-cell epitopes are targeted by effector memory T cells in HSV-seropositive asymptomatic individuals, indicating that this protein is prominently displayed to the adaptive immune system during infection [19]. Regarding cross-reactivity with heat-shock proteins, Zheng et al. demonstrated molecular mimicry between the HSV-1 tegument protein UL48 (VP16) and human heat-shock cognate protein Hsc71 [20]. Although direct evidence for UL46-HSP70 cross-reactivity is currently limited, the possibility of similar mechanisms involving other tegument proteins such as VP11/12 remains an intriguing area for future investigation. The upregulation of UL46 transcripts in circulating leukocytes may be associated with the broader immune dysregulation observed in BD.

UL29 stands out as the highest-performing diagnostic biomarker in this study. It encodes the major DNA-binding protein ICP8, which plays essential roles in viral replication and suppression of host antiviral responses [21, 22]. The robust overexpression of UL29 in BD patients, combined with its excellent ROC performance (AUC 0.90, specificity 92.5%), positions it as the strongest single blood-based biomarker identified in this study. The high specificity of UL29 is particularly clinically meaningful: it suggests strong potential as a “rule-in” test for BD. A positive UL29 result could support diagnostic confidence in ambiguous cases and help differentiate BD from other autoinflammatory or infectious conditions with overlapping mucocutaneous features. Prospective studies are warranted to evaluate whether UL29 transcript levels correlate with disease activity indices (e.g., BDCAF) and whether they normalize during remission.

The UL47-encoded tegument protein VP13/14 functionally cooperates with VP11/12 to modulate the trans-activation of viral immediate-early gene expression, and shares its lymphocyte-modulating properties [23]. VP13/14 has been further implicated in promoting post-translational modifications of host proteins that facilitate NLRP3 inflammasome assembly and caspase-1 activation, a pathway that converges on the maturation and secretion of IL-1β and IL-18 and the induction of pyroptosis [24, 25]. Hyperactivation of the NLRP3 inflammasome is a well-documented immunological hallmark of active BD; significantly elevated NLRP3, ASC, and caspase-1 expression levels have been demonstrated in peripheral blood mononuclear cells (PBMCs) and skin lesions of BD patients compared with healthy controls, and caspase-1 inhibition substantially attenuates IL-1β secretion in this context [26]. Despite this mechanistic rationale, UL47 exhibited the most modest diagnostic performance in the current cohort (AUC 69%, sensitivity 65%, *p* = 0.007). This discrepancy may reflect the broader involvement of inflammasome activation across diverse inflammatory conditions, reducing its disease specificity for BD. Alternatively, post-transcriptional regulatory mechanisms may decouple transcript abundance from functional VP13/14 protein levels. These considerations underscore that statistical significance alone is an insufficient criterion for biomarker selection; effect size, AUC, and likelihood ratios must be evaluated conjointly.

Finally, the transcriptomic findings reported here align with a growing body of multi-level evidence implicating HSV-1 in BD pathogenesis. Islam et al. demonstrated in a murine BD model that chronic psychological stress following HSV-1 inoculation significantly exacerbated the severity and frequency of BD-like inflammatory symptoms, providing controlled in vivo evidence that viral reactivation under physiological stress conditions can phenocopy the relapsing-remitting course of human BD [27]. Earlier human evidence was provided by Eglin et al., who detected HSV RNA in peripheral blood mononuclear cells of 73% of BD patients and 68% of those with recurrent oral ulcers, compared with only 12% of healthy controls (*p* < 0.001), using in situ nucleic acid hybridization — a methodologically distinct approach to the RT-qPCR transcriptomic profiling used in the present study [6]. At the serological level, Zheng et al. showed that serum IgG titers against the HSV-1 tegument protein UL48 were significantly higher in BD patients than controls (*p* < 0.01), and that anti-UL48 antibody levels correlated positively with disease activity indices (*r* = 0.48, *p* = 0.02); notably, further proteomics analysis revealed cross-reactivity between anti-UL48 antibodies and the human heat-shock cognate protein Hsc71, suggesting that molecular mimicry between viral tegument antigens and host chaperones may be a recurrent pathogenic theme in BD [20]. Taken together, these converging lines of evidence spanning murine models, human molecular virology, and serological profiling collectively reinforce the biological plausibility of the transcriptomic signature reported here.

Toward a multi-gene diagnostic panel and clinical translation, a consistent theme from our ROC analysis is that individual transcripts offer complementary strengths. While UL29 provides excellent specificity, combining it with UL46 (high sensitivity) and UL18 (intermediate profile) may yield additive diagnostic value. Although the current study did not conduct a combined ROC analysis, the non-overlapping performance characteristics of these three markers suggest that a weighted multi-gene panel could improve overall accuracy beyond that of UL29 alone. Future studies employing multivariable ROC or machine learning approaches are recommended to test this hypothesis formally. Such a panel, implemented via multiplex RT-qPCR, could offer a practical, cost-effective tool to support BD diagnosis and monitoring.

### Limitations and future directions

The present study has several limitations that should be acknowledged. Although the sample size of 80 participants (40 per group) was adequate for initial biomarker discovery, it remains relatively modest and restricts statistical power for subgroup analyses. Importantly, the lack of an independent validation cohort limits the reliability of the identified biomarkers. The study also did not include disease control groups (e.g., systemic lupus erythematosus, recurrent aphthous stomatitis, or other vasculitides), which makes it difficult to fully evaluate the specificity of the HSV-1 gene expression signature for Behçet’s disease. In addition, several clinically relevant variables were not available, including HSV serostatus, viral load, history of herpes episodes, HLA-B*51 genotyping, and medication profiles. These missing data represent potential confounding factors. The cross-sectional design further prevents causal inferences and does not allow assessment of how HSV-1 transcript levels change with disease activity, remission, or treatment response. Moreover, transcript-level changes do not necessarily reflect protein abundance or functional activity, as protein-level validation was not performed. Although GAPDH showed stable expression across samples, future analyses would benefit from normalization using multiple housekeeping genes. Finally, the single-center recruitment and ethnic homogeneity of the cohort may limit the generalizability of our findings. Despite these limitations, the results provide a solid foundation for future larger, multicenter, and prospective studies that include independent validation cohorts, appropriate disease controls, comprehensive clinical and virological data, and HLA-B*51 genotyping.

## Conclusion

This study demonstrates, in a well-characterized case-control cohort of 80 participants, that the HSV-1 transcripts UL29, UL18, and UL46 are significantly overexpressed in the peripheral blood of patients with active Behçet’s disease compared with healthy controls. These findings provide molecular support for the hypothesis that episodic or subclinical HSV-1 reactivation may contribute to BD immunopathogenesis through distinct but potentially complementary mechanisms: LILRB1-mediated NK-cell suppression and possible HLA-B*51 molecular mimicry (UL18), modulation of lymphocyte signaling pathways with potential HSP70 cross-reactivity (UL46), and attenuation of cGAS-STING-dependent innate immune sensing (UL29). From a translational perspective, UL29 emerged as the strongest individual biomarker, offering the highest AUC (90%) and excellent specificity (92.5%), which makes it particularly suitable for confirmatory or rule-in diagnostic applications. UL46 provided high sensitivity (87%), while UL18 offered intermediate performance. Together, these three transcripts show promise as a potential multi-marker panel. In contrast, UL47 exhibited more modest diagnostic utility. The additive value of combining UL29, UL46, and UL18 warrants formal evaluation using multivariable modeling in independent prospective cohorts. Future studies should aim to validate these findings at the protein level, explore the markers in less invasive samples such as saliva, and investigate the clinical benefit of antiviral prophylaxis in BD patients with evidence of HSV-1 reactivation. Such efforts will be critical for translating these observations into meaningful clinical tools.

## Data Availability

No datasets were generated or analysed during the current study.
